# First Evidence of *Rickettsia conorii* Infection in Dogs in Northern Tunisia

**DOI:** 10.3390/vetsci11090402

**Published:** 2024-09-01

**Authors:** Zeyneb Gharbi, Ahmed Ouni, Ghofrane Balti, Ali Bouattour, Ahmed Chabchoub, Youmna M’ghirbi

**Affiliations:** 1 National School of Veterinary Medicine, Institution of Agricultural Research and Higher Education, University of Manouba, Sidi Thabet 2020, Tunisia; ahmed.chabchoub@enmv.uma.tn; 2 Laboratory of Viruses, Vectors and Hosts (LR20IPT02), Institut Pasteur de Tunis, Université Tunis El Manar, Tunis 1002, Tunisia; ahmed.ouni@fst.utm.tn (A.O.); b.ghofraane@gmail.com (G.B.); ali.bouattour@pasteur.tn (A.B.)

**Keywords:** *Rickettsia conorii*, *Rhipicephalus sanguineus*, hematology, IFAT, real-time PCR, dogs, Tunisia

## Abstract

**Simple Summary:**

We conducted this study to investigate the role played by dogs in the spread of *Rickettsia* in Tunisia. Of the 136 dogs that were tested, 55.14% had anti-*Rickettsia conorii* antibodies while none had *Rickettsia* DNA. On the other hand, of 51 pools of *Rhipicephalus sanguineus* ticks collected from these dogs, 7 were *Rickettsia* DNA positive. These ticks contribute to the circulation of *Rickettsia*.

**Abstract:**

A cross-sectional study was carried out, between April 2021 and June 2022, to understand the role of dogs in the circulation of rickettsiosis in Tunisia. The presence of specific IgG antibodies against *Rickettsia conorii* was analyzed by indirect immunofluorescence test. By qPCR, blood and ticks were collected from 136 dogs examined at the Canine Department of National School for Veterinary Medicine of Tunisia. These dogs were also analyzed to detect *Rickettsia* DNA. The rate of *Rickettsia* seropositivity in 136 dogs was 55.14%. A total of 51 (53%) seropositive dogs showed clinical and biological signs such as fever and anorexia as well as thrombocytopenia and anemia. By qPCR, targeting the mitochondrial 16S rRNA gene, no *Rickettsia* DNA was detected in the blood. On the other hand, qPCR followed by sequencing revealed the presence of *R. conorii* subsp. *raoultii* in 7 tick pools of the 51 pools composed of the 227 ticks collected. A One Health approach to raise the awareness of dog owners to control tick infestations is imperative, given the dangers of canine zoonoses.

## 1. Introduction

Rickettsiosis is a zoonotic disease caused by several species belonging to the genus *Rickettsia* within the family Rickettsiaceae, order Rickettsiales. Considered as one of the oldest known vector-borne diseases, rickettsiosis has re-emerged as an important infectious disease affecting both humans and dogs worldwide [1].

In the Mediterranean region, *Rickettsia conorii*, an obligate intracellular gram-negative bacterium transmitted by ticks, is the major agent of the spotted fever group (SFG) in humans and in dogs [1,2]. *Rh. sanguineus* is the main vector and reservoir of *Rickettsia conorii* in regions with Mediterranean climates [3]. However, other tick species have been implicated as potential vectors, such as *Rh. turanicus* [4].

*R. conorii* was described in Tunisia in 1910 and is considered endemic [5]. Human clinical cases of rickettsial disease caused by *R. conorii* have been confirmed in several regions of Tunisia. The number of human cases is typically highest between June and October, coinciding with the peak tick season [6,7,8,9,10,11]. Over the past few decades, several studies have documented the presence of other *Rickettsia* species in Tunisia [6,7,8,9,10,11,12,13,14,15]. In particular, *R. conorii* subsp. *israelensis*, *R. conorii* subsp. *conorii*, *R. massiliae* have been identified in patients and in *Rhipicephalus sanguineus* ticks collected from dogs [9,10]. In addition, *R. aeschlimannii*, *R. helvetica* and *R. africae* have been reported in camel blood samples and in *Hyalomma dromedarii* ticks in southern and central Tunisia [13,14]. Similarly, *R. helvetica* and *R. monacensis* DNA have been found in *Ixodes ricinus* ticks [15]. In 2021, Belkahia et al. confirmed the presence of pathogenic *Rickettsia* spp. in *Rh. sanguineus* and *Rh. turanicus* collected from small ruminants [12].

Canine vector-borne diseases, particularly those transmitted by ticks, constitute a significant health problem for dogs worldwide because of abundance and environmental adaptability of vectors and climatic conditions [16,17,18]. The brown dog tick, *Rh sanguineus*, is a known vector for several tick-borne pathogens in dogs, including *Babesia* spp. and *R. conorii* [16,17]. In general, dogs that are naturally infected with *R. conorii* show no obvious clinical signs, although the infected dogs will seroconvert. Yet, some studies have reported the possibility of clinical disease in dogs infected by *R. conorii*, including fever and anemia [19,20,21]. Furthermore, febrile illness associated with *R. conorii* infection in dogs from Sicily [19] and Portugal [22], which exhibited anorexia and lethargy for 2–3 days, was reported. Levin et al. (2012) have also reported that most of the experimentally infected dogs had similar clinical symptoms, although the severity of symptoms varied depending on the bacterial strain, the mode of infection, and on the genetic background of the animals [23].

Dogs are the preferred host of *Rh. sanguineus*, serving as sentinels for *R. conorii* infection in humans. Indeed, because of their relationship with humans, dogs are considered the most effective sentinels and reservoirs for *R. conorii* [21]. Unfortunately, there is little available information in Tunisia on the occurrence of this pathogen in dogs. The aim of this study was to determine the role of dogs as a source of rickettsial spread through serological and molecular analysis. In parallel, ticks were collected from dogs for the detection of their infection with *R. conorii*.

## 2. Materials and Methods

### 2.1. Dogs and Blood Sampling

Between April 2021 and June 2022, a cross-sectional study was conducted to collect blood samples from dogs admitted to the Canine Department of National School for Veterinary Medicine of Tunisia for various reasons, including illness, vaccination, and sterilization. The medical records of the dogs were prospectively evaluated and information retrieved included age, sex, breed, tick infestation, signalment, residential area (urban vs. rural), lifestyle (mostly indoors vs. mostly outdoors), season (warm [May–September] vs. cold [October–April] seasons), clinical examination and hematologic abnormalities.

Blood samples were taken from the radial vein in 5 mL anti-coagulated tubes containing ethylene-diamine tetra-acetic acid (EDTA) for DNA extraction, and in 5 mL dry tubes for serological analysis.

### 2.2. Collection and Tick Processing

Ticks were carefully removed, with fine forceps, from different parts of the body of the admitted dogs and placed in labeled tubes containing 70% ethanol. A morphological identification of the ticks was conducted under a stereomicroscope (Leica wild M240, GmbH, Hilden, Germany) using taxonomic keys and tick guides [24,25]. Depending on their sex, stage and species, the ticks were grouped into pools of 1 to 7 specimens.

For the homogenization process, tick pools were rinsed with PBS and then with sterile water to ensure proper sample preparation and then transferred to collection tubes (Zymo Research, GmbH, Hilden, Germany) along with a 4 mm ceramic bead. Subsequently, 800 µL of AVL lysis buffer was added to each pool. Homogenization was performed using the Bead Raptor 24 homogenizer (Omni Bead Ruptor Elite, GmbH, Hilden, Germany) at a speed of 6.5 m/s for six 30 s cycles. The homogenates were then stored at −20 °C until DNA extraction.

### 2.3. Clinical Data and Cell Blood Count

Dogs were examined for different clinical signs such as fever, pyrexia, weakness, etc. A complete blood count was carried out using an automated laser flow cytometer unit (MINDRAY BC-2800, Guangzhou, China).

### 2.4. Serological Analysis

Anti-*Rickettsia conorii* IgG antibodies in the dog sera were analyzed using a commercial indirect fluorescent antibody test (IFAT) from Fuller Laboratories (Fullerton, CA, USA). Briefly, serum samples from each dog were tested at a dilution of 1:80 in phosphate-buffered saline. Positive and negative controls provided in the commercial kit were systematically included on each IFAT slide according to the manufacturer’s instructions (Fuller Laboratories, Fullerton, CA, USA).

### 2.5. DNA Extraction

DNA was extracted from both canine whole blood and homogenates tick pools using the QIAmp^®^DNA Mini kit (Qiagen GmbH, Hilden, Germany), following the manufacturer’s instructions. DNA samples were adjusted to a volume of 100 µL with the provided elution buffer (AE) and stored at −20 °C until further use. To assess the presence of contamination, a control tube containing distilled water was included in the extraction procedure for each of the samples. DNA yield was assessed using a NanoDrop ND-1000 spectrophotometer (NanoDrop Technologies, Wilmington, DE, USA).

### 2.6. PCR Amplification

Real-time PCR (qPCR) was used to investigate the presence of *Rickettsia* spp. DNA in both blood and tick samples. The qPCR used a set of primers and a probe that detects SFG species [26]. qPCR was performed on the ABI 7500 Real Time PCR system (Applied biosystems, Foster City, CA, USA). The reaction mixture, with a final volume of 20 µL, included 0.2 µM of each primer, 0.2 µM of probe and 12.5 µL of Premix ExTaq (Takara Bio Inc., Shiga, Japan) and 5 µL of DNA sample. After a hot-start cycle at 95 °C for 2 min, the reactions were cycled 40 times as follows: 95 °C for 15 s and 60 °C for 1 min.

### 2.7. DNA Sequencing and Data Analysis

To identify species detected in ticks, *gltA* gene was amplified and sequenced using primers previously reported [27]. PCR reactions were performed in a 25 µL reaction mixture containing 1 µM of each primer, 200 µM of each dNTP (Takara Bio Inc., Shiga, Japan), 0.75 U Taq polymerase (ExTaq, Takara Bio Inc., Shiga, Japan), 1X Taq buffer, and 5 µL extracted genomic DNA. Amplification was carried out in a thermocycler (Applied Biosystems, Hilden, Germany) under the following conditions: 10 min of initial denaturation at 94 °C, then 40 cycles of 94 °C for 1 min, 54 °C for 30 s, and 72 °C for 30 s. The amplification was completed by holding for 5 min at 72 °C to allow a complete extension of the PCR products. In each PCR, DNA of *R. montanensis* was included as a positive control and water as a negative control. The positive PCR products were purified using the ExoSAP cleanup procedure (Amersham Biosciences, Piscataway, NJ, USA). All nucleotide sequences were obtained using the Big Dye Terminator v.3.1 Cycle Sequencing Kit (Applied Biosystems, Foster City, CA, USA) and the 3130 automated sequencer (Applied Biosystems, CA, USA). The sequences generated in this study were edited and aligned using BioEdit v7.7.1.0 https://bioedit.software.informer.com/7.1/ (accessed on 20 May 2023) [28] and ClustalW software programs (CLUSTAL 2.0.12 Multiple Sequence Alignments) http://www.clustal.org/clustal2/ (accessed on 20 May 2023) [29]. The BLAST program http://www.ncbi.nlm.nih.gov/BLAST (accessed on 20 May 2023) was used to compare and analyze the data sequences.

### 2.8. Statistical Analysis

Statistical analysis was performed using IBM SPSS statistical software (Version 23.0, IBM Corp: Armonk, NY, USA). Descriptive methods were used to characterize the dogs sampled and the diagnostic test results (IFAT and PCR). Proportions were presented for categorical variables and 95% confidence intervals (CI) were estimated. Chi-square was used to test the associations between possible risk factors (breed, age, sex, season, lifestyle, and tick infestation) and the presence of the pathogen (PCR) and antibodies against *R. conorii* (IFAT). The differences were considered statistically significant with a *p*-value < 0.05. Binary logistic regression estimated the odds ratios (OR).

### 2.9. Nucleotide Sequences Data

Sequences data reported in this paper were deposited in GenBank database under the accession numbers OR 399543–OR 399549.

## 3. Results

### 3.1. Clinical Examination of Dogs

A total of 136 dogs aged 1 to 11 years were sampled (74 males, 62 females). The average age of the dogs was three years. A total of 39 of the 136 of dogs (28.67%) presented at least one clinical sign compatible with canine rickettsiosis, such as pyrexia, weakness, and fever.

### 3.2. Tick Infestation of Dogs

Ticks were collected from 39 dogs (39/136), resulting in an infestation rate of 24%. A total of 227 ticks were collected and identified as *Rhipicephalus sanguineus*. These consisted of 136 males, 79 females, and 12 nymphs, and were grouped into 51 pools.

### 3.3. IFAT Test

A total of 75 tested dogs (55.14%; 95% CI: 46.39–63.68%) presented IgG antibodies against *R. conorii.* Only 11 (8.1%; 95% CI: 4.11–14.01%) of the seropositive dogs presented clinical signs suggestive of canine rickettsiosis.

In female dogs, the seropositive rate of antibodies against *R. conorii* (59.68%; 95% CI: 46.45–0.71; 37/62) was higher than in males (51.35%; 95% CI: 39.44–63.15%; 38/74), although the difference was not statistically significant (*p* > 0.05) (Table 1).

The highest prevalence was observed in dogs aged between 2 and 7 years (46/65; 70.8%; 95% CI: 58.17–81.40), followed by those older than 7 (9/14; 64.3%; 95% CI: 35.14–87.24%). The lowest prevalence was observed in the youngest age categories (20/57; 35.09%; 95% CI: 22.91–48.87%). These differences were statistically significative (*p* < 0.0001) but the difference was not statistically significant for sex, breed, lifestyle, or tick infestation. On the other hand, a significant difference was observed in relation to the season (*p* = 0.009; Table 1).

The highest *Rickettsia* seropositivity (68%; 95% CI: 56.22–78.31%) was observed in sick dogs who showed various signs in clinical examination. The main clinical manifestations were fever (41.33%; 95% CI: 30.08–53.30%; OR 0.5978) followed by weakness (44.12%; 95% CI: 35.77–52.46%; OR 0.9894) and pyrexia (22.06%; 95% CI: 15.09–29.03%; OR 1.0822).

### 3.4. Hematological Alterations

A complete blood count was performed on only 119 dogs as the remaining 17 animals had volumes of sampling blood that were not sufficient to conduct this test. Among the seropositive dogs, 68 had hematological variations in their blood counts. Thrombocytopenia (reference value < 200 × 10^9^/L) was the most common hematological abnormality observed in 60% (95% CI: 48.04–71.15%) of seropositive dogs (45/75). Anemia (with reference values of RBC 5.5–8.5 × 10^12^/L; Hb 110–190 g/L; Ht 39–56%) is observed in 37.33% of cases (28/75; 95% CI: 26.43–49.27%). The seropositive rate of antibodies against *R*. *conorii* was associated with thrombocytopenia (*p* < 0.0001) and anemia (*p* < 0.0001).

### 3.5. PCR Analysis

All dogs (*n* = 136) tested by qPCR for detecting *Rickettsia* spp. DNA were negative, whereas among the 227 *Rh. sanguineus* ticks grouped into 51 pools, 7 pools proved to be infected by *Rickettsia* spp. (13.72%; 95% CI: 5.7–26.26%). Among 32 pools composed of females, 4 were positive, while of 14 pools composed of males, 3 were positive. The difference was not significant (*p* = 0.438). The five pools composed of nymphs were negative.

### 3.6. DNA Sequencing and Data Analysis

The seven qPCR positive pool ticks were sequenced to identify *Rickettsia* species targeting partially the *gltA* gene. A BLAST analysis of the obtained sequences revealed that all sequences were *Rickettsia conorii raoultii* with genetic variability in five nucleotide positions (406, 439, 444, 595, 922) (Table 2). The seven sequences (GenBank accession numbers OR 399543–OR 399549) showed significant identity (99.41–99.7%) with *R. conorii raoultii* sequences described in China (GenBank accession number MT178338, MF511249).

## 4. Discussion

In view of global changes, *Rickettsia conorii*, an agent of Mediterranean spotted fever (MSF), can pose a threat to human and animal health. Dogs can play an important role in the cycle of this bacteria. In this epidemiological study, a total of 136 dogs were included in an encompassed, serological, molecular, clinical, and hematological rickettsiosis investigation.

Using a commercial indirect immunofluorescence antibody test (IFAT), 55% of the scanned dogs were seropositive for *R. conorii*. This reveals the presence of anti-*Rickettsia* antibodies and exposure to the infection by this bacterium in dogs presented to the clinical department of the Sidi Thabet Veterinary School. The high overall seropositivity (55%) is not surprising, since in Mediterranean-endemic regions, *Rickettsia* seropositivity in dogs is still high. Indeed, similar results were observed in Italy, where clinically suspect dogs, mainly from northern and central Italy, tested by IFAT, showed a *Rickettsia* spp. seropositivity of 64.9% [17]. Likewise, in Sicily (Italy), 53.4% of tested dogs (*n* = 342) were seropositive for *R. conorii* [30]. In Greece, 46.5% of tested dogs (93/200) had antibodies to *R. conorii* [31]. In Montenegro, Lauševi et al. (2019) reported a *R. conorii* seropositivity of 81.9% in domestic dogs [32]. In Portugal, with IFA test, Alexandre et al. (2017) revealed that 62% of dogs suspected of having a tick-borne illness and 38.5% of healthy dogs had IgG antibodies reactive with *R. conorii* [22]. In northern Spain, 13.7% of tested dogs (10/73) had antibodies to *R. conorii* [33]. Seropositivity was 1.6% and 44.8% in dogs from Croatia and Serbia, respectively [34,35]. More recently, in northern Portugal, a lower seropositivity was recorded (9.7% of 113 tested dogs) [36]. Overall, these findings show that the seropositivity of *Rickettsia* in dogs differs between regions with different risk factors. Indeed, in our study, we recorded significantly higher seropositivity in animals older than two than those less than a year old. This result is in agreement with Mendoza-Roldan et al. (2021) reporting that the seropositivity of *Rickettsia* spp. varied significantly according to age [17]. This higher seropositivity reported in the oldest dogs is closely associated with prolonged exposure to infected ticks. In contrast, in Portugal, Afonso et al. (2024), noted that age was not associated with seropositivity of *R. conorii* [36].

The seropositive rate of antibodies against *R. conorii* did not differ between males and females. A similar result was reported in Thailand [37], while other studies have shown that seroprevalence is sometimes higher in males [38] and sometimes in females [36]. Indeed, dogs roaming more and engaging in outdoor activities are more likely to be exposed to tick infestations, which increases the risk of *Rickettsia* infection.

The present study shows also that seropositivity is higher during the warm season, which corresponds to the activity of tick vectors in Tunisia, notably *Rh. sanguineus* [39]. This is consistent with the results of Ortuno et al. (2009) who reported that seropositivity was higher in dogs highly exposed to *Rh. sanguineus* [40]. It is known that anti-*R. conorii* antibodies in naturally infected dogs have a short life and that the prevalence and antibody titers decrease rapidly in dogs in winter (cool season) when ticks are inactive but increase again once tick activity resumes [23,40]. Otherwise in Tunisia, the presence of ticks on dogs has become permanent all year round, which could be attributed to global warming. We did not record a significant correlation between seropositivity with the infestation of dogs by ticks (*p* = 0.274). This does not exclude the fact that these dogs were previously exposed to infected ticks.

In our study, to screen *Rickettsia* seropositive dogs, we used the IFA test that shows a sensitivity greater than 95%. However, this test shows limitations due to cross-reactions. To overcome this drawback, we used qPCR technique to detect *Rickettsia* DNA in the blood of the 136 admitted dogs, but the results show that these animals were all qPCR negative. The discrepancy between serological and qPCR results is not uncommon and can be attributed to several factors. Indeed, this finding can be attributed to low amount of *Rickettsia* organisms *Rickettsia* circulating in the blood and probably rapidly cleared due to the immunocompetence of the dog, which develops protective humoral immunity [19]. Indeed, Levin et al. (2012) have shown, by experimental infections of dogs with *R. conorii*, that rickettsiemia is very short-lived [23]. To the contrary, in several studies, the DNA of *Rickettsia* was detected in sick dogs. Indeed, in southern Italy, Solano-Gallego et al. (2015) reported that 3% of 99 dogs with acute fever are *Rickettsia* PCR positive [21]. Similar results were observed in Portugal and in Angola with ill dogs [22,41]. It is thus interesting to note that studies describing infected dogs have reported that animals show severe symptoms, whereas the dogs of the present study show no serious signs of disease. These studies point to the potential role of dogs as a reservoir and sentinel host [21]. The experimental *Rickettsia* infections of dogs, conducted by Levin et al. (2012) confirmed these hypotheses as they proved that dogs infected by *R. conorii israelensis* could transmit this pathogen to new groups of uninfected ticks [23].

Clinical manifestations of *R. conorii* infection in dogs vary from subclinical to commonly mild disease [42]. In our study, 96 of 136 dogs (70.6%) presented at least 1 clinical sign compatible with canine rickettsiosis, such as anorexia, weakness and fever. In fact, signs of spotted fever rickettsioses in dogs are not specific [43]. In general, *R. conorii* can infect dogs, causing fever and other tick-borne unspecific symptoms such as the acute onset of fever, and lethargy [21]. Furthermore, among sick examined dogs, only 51 (53%) were *Rickettsia* seropositive, the remaining (*n* = 24) were healthy. *Rickettsial* infections may be asymptomatic in some dogs even after seroconversion [44]. This is in agreement with Mannelli et al. (2003) who concluded that healthy dogs are commonly seropositive in endemic regions [2].

In addition, seropositive dogs with clinical signs were associated with hematological abnormalities. The most prominent hematological changes were thrombocytopenia and anemia. Our result confirms the study of Solano-Gallego et al. (2015) who deduced that the presence of an initial high *R. conorii* antibody titer was statistically associated with thrombocytopenia and anemia [21].

Only 24% of the admitted dogs were infested with ticks, and all the ticks that were removed were identified as *Rh. sanguineus*. This low infestation rate may reflect the fact that the studied dogs are mostly well maintained. In dogs randomly chosen in Tunisia, M’ghirbi and Bouattour, 2008 reported an infestation rate of 70% [39]. In general, stray dogs show a high prevalence of infestation with *Rh. sanguineus* that can reach up to 100% in some regions [45]. This three-host tick, which is well adapted to human environments, is considered to be the main reservoir of *R. conorii* [23]. Using qPCR, we revealed the presence of *R. conorii* subsp *raoulti*, in seven pools of *Rh. sanguineus*. In a previous study, Khrouf et al. (2014) reported *R. conorii* subsp *israelensis* (spotted fever strain) in *Rh. sanguineus* collected from dogs in southern Tunisia [8]. However, in Algeria and Morocco, by PCR, *Rh. sanguineus* ticks were positive for *R. massiliae* (spotted fever group) [46]. 

*Rickettsia* spp. infections in *Rh. sanguineus* contribute to a higher risk of transmission of these pathogens to human. Indeed, by molecular techniques, studies have revealed the presence of *R. conorii* and *R. massiliae* in sick patients in Tunisia where several human cases were recorded every year [10].

## 5. Conclusions

In Tunisia, global warming is lengthening the warm season (March–October), which has favored the exposure of dogs to tick infestation and consequently to tick-borne diseases such as rickettsiosis. The DNA of *R. conorii* was revealed in ticks collected from dogs brought to the school of veterinary medicine. In total, 55.14% of these dogs had anti-*Rickettsia* antibodies, with some showing clinical signs and hematological abnormalities. Since *R. conorii* is responsible for several human cases, the effective control of ticks on dogs and in the environment constitute an effective issue to prevent human and animal rickettsiosis in the One Health context.

## Figures and Tables

**Table 1:** Seropositive rate of *Rickettsia conorii* in dogs according to the risk factors.

Risk Factor	Nb of Seropositive Dogs/Nb of Tested Dogs (%) [95% CI]	OR (95% CI)	*p*-Value
Age (year)			
<1	20/57 (35.1) [22.91–48.87]	Reference	<0.0001
[2–7]	46/65 (70.8) [58.17–81.40]	1.820 (1.068–3.101)	
>7	9/14 (64.3) [35.14–87.24]	1.353 (0.632–2.897)	
Sex			
Male	38/74 (51.35) [39.44–63.15]	Reference	0.330
Female	37/62 (59.68) [46.45–71.95]	1.40 (0.7–2.77)	
Breed			
Watch dogs	59/109 (54.13) [44.32–63.71]	Reference	0.523
Hunting dogs	9/13 (69.23) [38.57–90.91]	1.625 (0.692–3.818)	
Pet dogs	7/14 (50) [23.04–76.96]	0.722 (0.322–1.619)	
Tick infestation			
Infested	16/34 (47.06) [29.78–64.87]	Reference	0.274
Non-infested	59/102 (57.84) [47.66–67.56]	1.54 (0.7–3.36)	
Lifestyle			
Confined	61/111 (54.95) [45.22–64.41]	Reference	0.924
Outdoor access	14/25 (56) [34.93–75.60]	1.04 (0.43–2.49)	
Season			
Cold	4/16 (25) [7.27–52.38]	Reference	0.0098
Warm	71/120 (59.17) [49.82–68.05]	1.96 (1.31–2.30)	

**Table 2:** *R. conorii* sequencing analysis results.

GenBank Accession Number	Blast Analysis	Similarity (%)	Host (Country)	Nucleotide Positions *				
				406	439	444	595	922
OR 399543	MF511249	99.54	*Ixodes persulcatus* (China)	A	C	A	C	G
OR 399544	MT178338	99.41	*Dermacentor nuttalli* (China)	A	G	A	T	G
OR 399545	MT178338	99.41	*Dermacentor nuttalli* (China)	A	G	A	T	G
OR 399546	MT178338	99.41	*Dermacentor nuttalli* (China)	A	G	A	T	G
OR 399547	MT178338	99.41	*Dermacentor nuttalli* (China)	A	G	A	T	G
OR 399548	MT178338	99.54	*Dermacentor nuttalli* (China)	T	G	G	A	A
OR 399549	MF511249	99.7	*Ixodes persulcatus* (China)	T	G	G	A	A

* Nucleotide positions are indicated referring to the complete *gltA* gene sequence.

## Data Availability

Sequences data reported in this paper were deposited in GenBank database under the accession numbers OR 399543–OR 399549.
